# In Vitro Study to Evaluate the Best Conditions Highlighting the Antimicrobial Activity of *Carum carvi* Essential Oil on Human Pathogen Isolates in Formulations Against the Spread of Antibiotic Resistance

**DOI:** 10.3390/ph18030321

**Published:** 2025-02-25

**Authors:** Carolina Mastrella, Silvia Rizzo, Maura Di Vito, Stefania Garzoli, Mattia Di Mercurio, Melinda Mariotti, Marilena La Sorda, Abdesselam Zhiri, Maurizio Sanguinetti, Francesca Bugli

**Affiliations:** 1 Dipartimento di Scienze Biotecnologiche di Base, Cliniche Intensivologiche e Perioperatorie, Università Cattolica del Sacro Cuore, 00168 Rome, Italysilvia.rizzo@unicatt.it (S.R.); mattia.dimercurio@unicatt.it (M.D.M.); melinda.mariotti@unicatt.it (M.M.); maurizio.sanguinetti@unicatt.it (M.S.); francesca.bugli@unicatt.it (F.B.); 2 Dipartimento di Chimica e Tecnologie del Farmaco, Università di Roma Sapienza, Piazzale Aldo Moro 5, 00185 Rome, Italy; stefania.garzoli@uniroma1.it; 3 Dipartimento di Scienze di Laboratorio e Infettivologiche, Fondazione Policlinico Universitario A. Gemelli IRCCS, Largo A. Gemelli 8, 00168 Rome, Italy; marilena.lasorda@policlinicogemelli.it; 4 R&D Department, Pranarom International, 37 Avenue des Artisans, 7822 Ghislenghien, Belgium; azhiri@pranarom.com; 5 Plant Biotechnology, Université Libre de Bruxelles (ULB), CP300, Rue Prof. Jeener & Brachet 12, 6041 Gosselies, Belgium

**Keywords:** broth microdilution test, disk diffusion test, microatmosphere test, Tween80, DMSO, ethanol, antimicrobial resistance

## Abstract

**Background/Objectives**: In recent years, antimicrobial resistance has become a major threat to global health, and scientific research aiming to identify new therapeutic resources is a priority. Essential oils (EOs), obtained from spices belonging to the culinary tradition, like *Carum carvi* essential oil (CC-EO), are of great interest for their antimicrobial activity, but the methods used to evaluate their efficacy need to be standardized. The aims of this work were to evaluate the following: (i) the best microbiological in vitro test; (ii) the best surfactant; and (iii) the best microbiological target of CC-EO and its method of administration. **Methods**: CC-EO quality was evaluated using gas chromatography–mass spectrometry. Antimicrobial susceptibility testing with drugs currently in use was performed. Antimicrobial effectiveness against 70 clinical strains belonging to *S. aureus*, *E. coli*, *E. faecalis*, *K. pneumoniae*, *P. aeruginosa*, *S. pyogenes*, and *C. albicans* was evaluated. Two microbial tests (broth microdilution tests and disk diffusion), generally used in routine clinical practice, were compared. To choose the best vehicle, Tween80, DMSO, and ethanol were evaluated. The antimicrobial efficacy of vapors was assessed using a microatmosphere test. **Results**: The broth microdilution test is confirmed as the best in evaluating the antimicrobial activity of EOs. The most suitable EOs vehicle for antimicrobial testing was Tween80. CC-EO and its vapors were effective against GRAM+ and *C. albicans* strains, both sensible and resistant, and ineffective against GRAM−. **Conclusions**: In the future, it may be possible to include CC-EO in topical or spray formulations for the treatment of GRAM+ and *C. albicans* infections.

## 1. Introduction

In the last few years, antibiotic resistance has become a global health emergency. Antibiotic resistance is a natural biological adaptation phenomenon for some microorganisms; they acquire the ability to survive or grow in the presence of a concentration of antimicrobial drug generally sufficient to inhibit or kill microorganisms. The loss of effectiveness is due to microorganisms implementing strategies to survive by altering their genetic makeup, especially in the presence of these drugs. The acquisition of resistance is significantly facilitated by lateral gene transfer (LGT), which enables the exchange of resistance genes between different bacterial species [1].

Bacterial resistance manifests through various mechanisms, including the expulsion of the antibiotic through the efflux pumps, reducing the concentration of the antibiotic and preventing antibiotic entry by altering cell membrane permeability. Resistance can also occur through modifying the antibiotic molecules to render them ineffective, such as through the production of beta-lactamases, i.e., enzymes that break down beta-lactam antibiotics, and through decreasing an antibiotic’s affinity for its target by mutating or altering molecular targets [2].

Although new formulations have been introduced over the last 30 years, progress in developing new drugs remains insufficient while the phenomenon of antibiotic-resistance is spreading exponentially. New approaches must be found quickly as if no changes are imposed, projections show that in 2050, about 10 million deaths related to infections caused by multi-resistant germs will be reached [3].

Data from antibiotic resistance studies have highlighted the severity of the issue. Between 2016 and 2020, the annual number of cases of infections—including by resistant strains such as *Escherichia coli*, *Staphylococcus aureus*, *Enterococcus faecium*, *Pseudomonas aeruginosa*, *Klebsiella pneumoniae*, and *Acinetobacter baumannii*—in the EU/EEA ranged from 685,433 (95% Uncertainty Interval UI 589,451–792,873) in 2016 to 865,767 (95% UI 742,802–1,003,591) in 2019, with an annual number of attributable deaths ranging from 30730 (95% UI 26935-34836) in 2016 to 38,710 (95% UI 34,053–43,748) in 2019 [4]. The prevention and treatment of infections, especially bacterial ones, lead to a reduction in mortality and therefore achieve health equity. In parallel, resistance to antifungal agents, like antibiotic resistance, is a growing concern [5]. Fungal infections affect over 1 billion people globally, with approximately 150 million suffering from severe infections that significantly compromise their quality of life or lead to death. Unfortunately, the antifungal drugs group is not as large as that of antibiotics, and this greatly reduces the number of therapeutic weapons that can be wielded against fungal infections. This limitation, together with the limited availability of antifungal agents, exacerbates the development of drug resistance. Despite advancements in antifungal treatments, the mortality rate remains high, reaching approximately 40% [6].

In this context, developing a wide range of therapeutic alternatives is crucial. The prevention and management of resistance require multidisciplinary approaches, together with the development of new methodologies, including scientific and practical strategies. In this scenario, the adoption of a One Health approach is essential to prevent and reduce healthcare-associated infections, thereby minimizing the spread of resistance [7].

Medicinal plants, and the natural substances extracted from them, have always been an important resource that humanity has studied and exploited to identify new resources useful for preserving health. In Europe, when a medicinal plant shows at least 30 years of popular use, and 15 in any of the Member Countries, its drug and extracts are understood to have been fully incorporated into the remedies used in traditional medicine [8].

In recent years, innovative alternatives to traditional antimicrobials have been gaining attention. Among these, essential oils (EOs) have emerged as a promising solution [9].

EOs are plant-derived compounds, consisting of mixtures of active volatile substances produced by the secondary metabolism of plants, generally obtained through distillation, except for plants belonging to the *Citrus* genus. These EOs, primarily composed of terpenes, exhibit broad-spectrum antimicrobial activity. Their complex chemical composition allows EOs to act mechanically (i.e., via disruption of the membrane/cell wall) and chemically (i.e., via interference with cellular metabolism), minimizing the risk of developing resistance. In this way, the microorganism is forced to fight on multiple fronts against EOs, which often prevail by inhibiting or destroying it [9]. Indeed, even if EOs’ activities are still under study, it can be assumed that their principal mechanisms of action are non-specific and/or mechanical ones. For this reason, the likelihood of infections developing resistance to EOs is very limited, and they can also be active against multi-resistant germs [10,11].

Furthermore, EOs can be used not only with respect to human pathologies but also—and especially—in the livestock, crops, and food sectors to decrease the number of microorganisms, as well as with respect to public environments and surfaces to prevent the spread of pathogenic strains and reduce the use of antimicrobials [12].

Some characteristics, such as lipophilicity, volatility, and susceptibility to oxidation, make the use of EOs more complicated than synthetic drugs. For these reasons, it is necessary to carefully evaluate the mixtures, administration, and storage methods of EOs. However, the application of nanotechnology has opened new perspectives, allowing many of these limitations to be overcome [13].

For this study, the *C. carvi* EO (CC-EO) is chosen for its broad pharmacological properties. The CC aromatic plant belongs to the Apiaceae family. It is a biennial plant native to northern Europe, central-western Asia, and northern Africa. CC-EO shows a carminative property and is commonly used to treat digestive diseases such as flatulence, dyspepsia, and heartburn. CC-EO is rich in monoterpenes that contribute to its strong antimicrobial activity against GRAM+ and GRAM− bacteria, as well as various fungi [14,15,16,17]. The antimicrobial effectiveness of CC-EO has been widely studied against plant pathogens. Liu C. et al. highlight CC-EO for its antifungal and antiflatoxigenic effects against *Penicillium* species, including *P. brevicompactum*, *P. citrinum*, *P. crustosum*, *P. expansum*, *P. funiculosum*, *P. glabrum*, *P. chrysogenum*, *P. oxalicum*, *P. polonicum*, and *Talaromyces purpurogenus*, using the disk diffusion method. Moreover, other studies—these using the agar diffusion method—have reported the antibacterial activity of CC-EO against GRAM+ and GRAM− bacterial species such as *Clavibacter*, *Curtobacterium*, *Rhodococcus*, *Erwinia*, *Xanthomonas*, *Ralstonia*, and *Agrobacterium*, which cause diseases in plants and cultivated mushrooms worldwide [18]. Additionally, CC-EO has shown antimicrobial activity against various microorganisms, including *Agrobacterium*, *Sitophilus oryzae* L., *Aspergillus flavus*, *Clavibacter*, and *Vibrio* spp. While the antimicrobial properties of CC-EO have been explored, there is limited evidence regarding its activity against human pathogens. Changhai Liu et al. demonstrate the efficacy of CC-EO against methicillin-resistant *Staphylococcus aureus* (MRSA), showing an inhibitory effect against planktonic bacteria and biofilm [19].

In our study, we broadened the range of human pathogens species analyzed. Specifically, the antimicrobial properties of CC-EO against ten bacterial and fungal strains, belonging to seven different species—*S. aureus*, *E. coli*, *E. faecalis*, *K. pneumoniae*, *P. aeruginosa*, *S. pyogenes*, and *C. albicans*—was investigated. Our study focused on strains isolated from clinical samples that ensure greater relevance for medical application. Among the analyzed strains, many exhibit antibiotic resistance profiles, for which there are scarce therapeutic alternatives that need to be found. Several methodologies commonly employed in the literature for the analysis of the antimicrobial activity of EOs [20] and their interaction with various vehicles (Teewn80, Dimethyl sulfoxide DMSO, and absolute ethanol) were analyzed. The comparison allows us to evaluate the best microbiological test to identify the antimicrobial activity of CC-EO and to define the best method of administration of the CC-EO (by nebulization or contact). The above is aimed at better understanding the properties of CC-EO obtained by one of the spices widely used in the Italian and Mediterranean culinary tradition, all to identify new applications that can counteract, from a One Health perspective, the development of antibiotic resistance.

## 2. Results

### 2.1. Clinical Isolates Characterization

The bacterial and yeast collection belonging to clinical strains were isolated from monomicrobial-positive clinical cultures. The antimicrobial resistance (AMR) profiles of the strains are included in Supplementary Materials Table S1a–g. According to Magiorakos A.P. et al., the bacteria methicillin-resistant *S. aureus* MRSA, *S. aureus*, *E. coli*, *E. faecalis*, *K. pneumoniae*, *S. pyogenes*, and *P. aeruginosa* non-susceptible to ≥1 agent in ≥3 antimicrobial categories are defined as multi-drug resistant (MDR) [20]. According to Arendrup et al., for *Candida* strains, *C. albicans* non-susceptible to ≥1 agent in ≥3 antimicrobial categories is defined as MDR [21].

### 2.2. Gass Chromatography–Mass Spectrometry

Gass chromatography–mass spectrometry (GC-MS) analysis allowed for the detection and identification of the sixteen components listed in Table 1. 

The chemical composition was dominated by components belonging to the monoterpene family. Carvone (65.1%) and limonene (31.9%) were the main components, followed by several minor compounds whose mean percentage values ranged from 0.1% to 0.9%.

### 2.3. Antimicrobial Activity

#### 2.3.1. Broth Microdilution Assay

Table 2 shows the results of the in vitro broth microdilution susceptibility tests using Tween80 as the vehicle for the essential oil.

The results obtained with broth microdilution susceptibility tests do not demonstrate any difference in susceptibility between the resistant and susceptible strains of each group. Among bacterial strains, *P. aeruginosa* exhibited the highest resistance, with MIC values ≥ 72.8 × 10^3^ μg/μL for most isolates. Notably, MICs values of *S. pyogenes* and *C. albicans* are lower compared to the other tested strains. Finally, *C. albicans* shows the low variability of all tested strains.

#### 2.3.2. Disk Diffusion Assay

**Figure 1:**
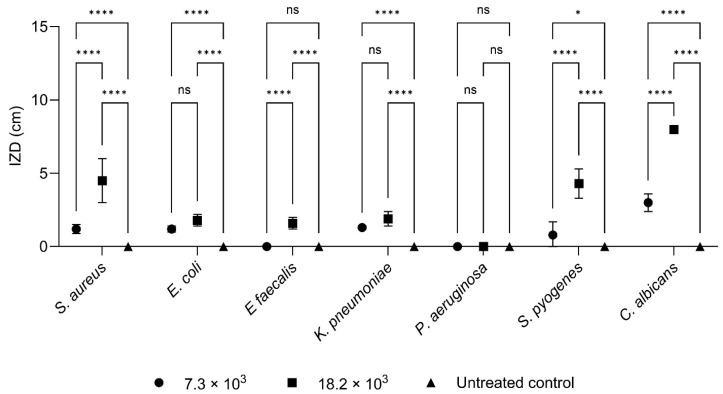
Graphical representation of the growth inhibition zone diameter (IZD) produced by 7.3 × 10^3^ μg and 18.2 × 10^3^ μg of CC-EO against *S. aureus*, *E. coli*, *E. faecalis*, *P. aeruginosa*, *K. pneumoniae*, *S. pyogenes*, and *C. albicans*. Statistical significance of the differences is indicated by * (*p* < 0.005), **** (*p* < 0.0005) and ns (*p* > 0.05), as shown in the figure.

**Figure 2:**
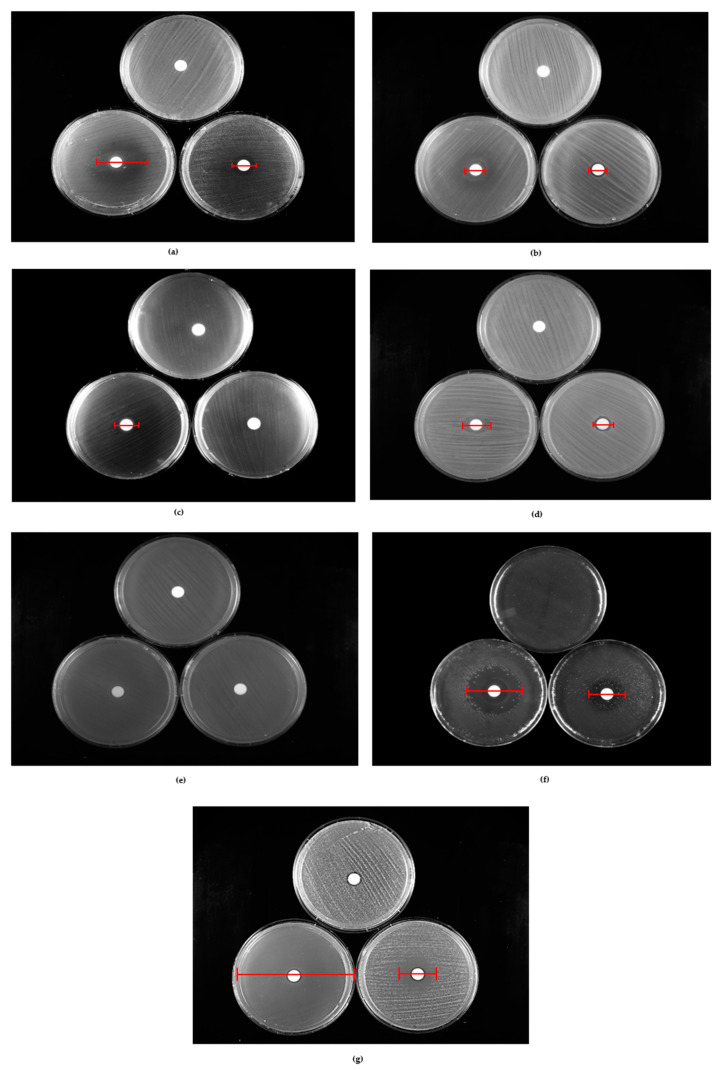
Representative images of growth IZD produced at two tested concentrations of *C. carvi* essential oil (by 7.3 × 10^3^ μg and 18.2 × 10^3^ μg) against *S. aureus* (**a**), *E. coli* (**b**), *E. faecalis* (**c**), *P. aeruginosa* (**d**), *K. pneumoniae* (**e**), *S. pyogenes* (**f**), and *C. albicans* (**g**). The red lines indicate the diameter of the inhibition halo. Each image includes three plates: a positive control of growth with the vehicle only; a sample treated with 18.2 × 10^3^ μg on the left; and a sample treated with 7.3 × 10^3^ μg on the right.

The results of the in vitro disk diffusion tests are shown in Figure 1 and Figure 2, Table 3, and Supplementary Materials Table S2.

*C. albicans* was the most susceptible microorganism, achieving maximum inhibition (8.0 cm) at the higher quantity of CC-EO tested (18.2 × 10^3^ μg). *P. aeruginosa* exhibited complete resistance, with no measurable inhibition at either concentration. *S. aureus* demonstrated the most notable increase in the IZD among the tested concentrations, highlighting a dose-dependent effect.

#### 2.3.3. Vehicles Test

A broth microdilution assay was used to evaluate the effectiveness of three different vehicles in dissolving the EO in a liquid medium. The vehicles tested were Tween80, DMSO, and ethanol. Two strains per species were tested: one with a resistant antimicrobial profile (R) based on susceptibility testing; and the other with a sensitive profile (S). According with the sensitivities shown in Table S1, the strains selected were as follows: 1^(R)^ and 6^(S)^ for *S. aureus*; 1^(S)^ and 9^(R)^ for *E. coli*; 2^(R)^ and 4^(S)^ for *E. faecalis*; 1^(S)^ and 8^(R)^ for *P. aeruginosa*; 2^(S)^ and 10^(R)^ for *K. pneumoniae*; 2^(R)^ and 3^(S)^ for *S. pyogenes*; and 2^(S)^ and 5^(R)^ for *C. albicans*. Data showed that MIC_average_ values are lower in five out of seven groups when using Tween80 instead of DMSO, and in four out of seven groups when compared to ethanol (Table 4).

#### 2.3.4. Microatmosphere Assay

**Figure 3:**
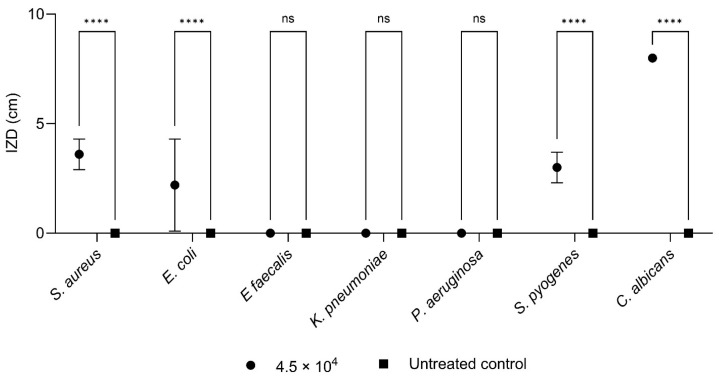
Graphical representation of the growth inhibition zone diameter (IZD) produced by 4.5 × 10^4^ μg of CC-EO against *S. aureus*, *E. coli*, *E. faecalis*, *P. aeruginosa*, *K. pneumoniae*, *S. pyogenes*, and *C. albicans*. Statistical significance of the differences is indicated by **** (*p* < 0.0005) and ns (*p* > 0.05), as shown in the figure.

**Figure 4:**
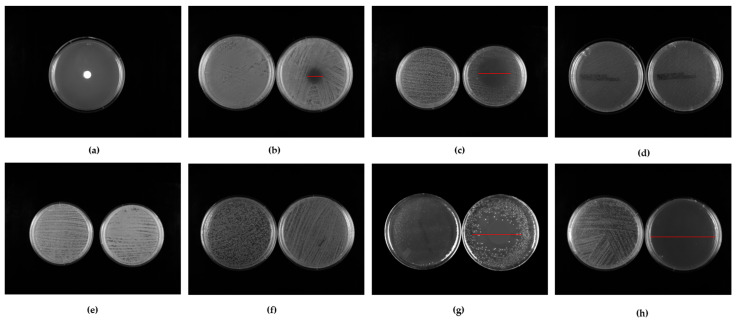
(**a**) Model plate. Representative images of growth inhibition zone diameter (IZD) produced at 4.5 × $10^{4}$ μg of CC-EO against *S. aureus* (**b**), *E. coli* (**c**), *E. faecalis* (**d**), *P. aeruginosa* (**e**), *K. pneumoniae* (**f**), *S. pyogenes* (**g**), and *C. albicans* (**h**). Each image includes an untreated sample on the left and a treated sample on the right.

IZDs values obtained by a microatmosphere test using 4.5 × 10^4^ μg of CC-EO against 70 isolates (10 strains per species) are summarized in Figure 3 and Figure 4, Table 5, and in Supplementary Materials Table S3.

*E. faecalis*, *K. pneumoniae*, and *P. aeruginosa* show IZDs of 0.0 mm; they are completely resistant to the tested concentration under microatmospheric conditions. *C. albicans* was the most susceptible microorganism, with uniform IZDs of 8.0 mm across all strains. Among bacteria, *S. aureus* shows the highest sensitivity to EO vapors, with an IZD_Average_ of 3.6 ± 0.7 cm, followed by *S. pyogenes* (3.0 ± 0.7 cm) and *E. coli* (2.2 ± 2.1 cm).

## 3. Discussion

In 2015, the European Medicine Agency (EMA) published the assessment report related to the fruit of *C. carvi* and its *aetheroleum* [22], classifying this spice and its extracts as part of traditional medicine. The medical use of this spice was first reported in European medical manuals in 1938 by Madaus Gerhard. To benefit from the CC’s carminative activity, this German doctor, in one of his three volumes of traditional medicine, reported the use of three teaspoons (=12 g) of CC-fruit in a glass containing hot water to drink during the day [23]. The ethnomedical use of CC-fruit has spread from Northern Europe to the Mediterranean region, Russia, Iran, India, Indonesia, and North America [24].

CC-EO is extracted by the hydro-distillation of CC-fruits which, according to the European Pharmacopeia, must contain a minimum of 30 mL/kg of EO [25] CC-EO is characterized by 50–65% of carvone and up to 45% limonene. The GC-MS analyses of the CC-EO used in this study confirm those provided by the manufacturer (see Supplementary Materials Table S4) and are in line with what is reported in the literature. The EMA document reports the use of dried fruit and its EO as carminative for the treatment of gastrointestinal problems, flatulence, and bloating. The daily dose of CC-EO reported in the European Union herbal monograph varies depending on the type of administration. Specifically, it is equal to 0.15–0.3 mL of EO divided into 1–3 doses for oral use and in semi-solid preparations at 2% for abdominal topical applications [22]. The chemical profile of CC-EO used in this study is in line with the chemical characteristics reported in the literature as it shows carvone and limonene as the main terpene components (Table 1).

Recently, in addition to the carminative activity, the scientific community is also paying attention to the antimicrobial effectiveness of EOs extracted from the genus *Carum*. This type of scientific study arises from the need to identify new sources with antimicrobial activity, but most studies were conducted using the EO extracted from *Carum copticum.* From 2002 to today, only about ten studies have been published on PubMed on the antimicrobial activity of CC-EO. The first of these studies was published in 2005 by an Italian research group led by Iacobellis N.S. [17], while the others have all been published in the last five years. Most of these studies explore the antimicrobial activity of CC-EO against pathogens of plants and/or environment, and only a few studied its activity against clinical strains [18].

This study evaluates the antimicrobial activity of CC-EO on a total of 70 strains obtained from seven species of clinical interest. The clinical analysis that explores the strains’ sensitivity against antimicrobial drugs shows that the percentage of resistant strains do not represent the Italian incidence detected by the National Surveillance of Antibiotic Resistance (AR-ISS) in 2024 (see Supplementary Materials). This difference is explained by the sample group being too small, taken in a single period of the year, and limited to a single hospital center; therefore, it is inappropriate to use this sample to represent the Italian population. In any case, the sensitivity of microbial strains towards CC-EO does not highlight statistically significant variations between the MIC values detected within each microbial population. This indicates that CC-EO behaves in the same way on drug-sensitive and drug-resistant strains.

The sensitivity of microbial strains against CC-EO was evaluated using the two most popular tests in clinical microbiology: the broth microdilution test and the agar diffusion test. The broth microdilution test is considered the “gold standard” in the study of the antimicrobial activity of EOs; whereas the agar diffusion test is widely used in both the scientific literature and clinical practice but is currently considered inappropriate due to several limitations that make the test unsuitable for EOs [25]. Therefore, this study not only evaluated the antimicrobial activity of CC-EO but also provided a comparison between two clinical analysis methods.

As shown in Table 2, using the broth microdilution test, CC-EO showed its maximum activity against strains of *S. pyogenes* and *C. albicans*, with MIC_average_ values of 4.0 × 10^3^ μg/mL and 5.5 × 10^3^ μg/mL, respectively; whereas the weakest activity was observed against *P. aeruginosa* strains, which, in most cases (6 out of 10 strains), did not show sensitivity to the concentrations tested. Furthermore, only *E. coli*, *E faecalis*, and *C. albicans* strains showed more punctual CC-EO activity, as 90% of the strains showed a difference between MIC50 and MIC90 not exceeding one dilution. On the contrary, the remaining strains showed differences between these two values equal to or greater than two dilutions, indicating a wider range of efficacy and therefore greater variability. The data shown in this article add further information to the traditional oral use widely documented in EMA monographs. In fact, the in vitro antimicrobial activity shows the effectiveness of CC-EO when placed, at the MIC concentrations, in direct contact to microbial strains. The absence of pharmacokinetic data in animal models complicates the translation of in vitro findings into a potential antimicrobial systemic application of the CC-EO. Instead, if further specific studies will confirm the safety of the in vitro data, it will be possible to use CC-EO in topical antimicrobial mucocutaneous use, including aerosol administration for the oral cavity.

Similarly, with the agar diffusion test, the strains showing low sensitivity were those of *P. aeruginosa*, which did not show any inhibition zone, and *E. faecalis*, which showed the lowest mean inhibition diameter. On the contrary, the most sensitive strain was that of *C. albicans*, which is totally inhibited using the maximum volume of EO tested (1.8 × 10^3^ mg), followed by *S. aureus* and *S. pyogenes* (Table 3). However, the major difference between the methods concerns the amount of CC-EO able to completely inhibit the growth of the strains. This difference is clearly visible when analyzing the values obtained with *C. albicans* strains that show a total growth inhibition (i.e., one equal to the diameter of the Petri dish), exposing the strains to an amount of EO approximately 2 × 10^3^ times higher than the MIC90 obtained with the broth microdilution (9.1 mg/mL).

MIC values obtained with the agar diffusion method lead specialists to underestimate the efficacy of CC-EO against microbial strains. This error is determined by critical issues related to the method. Specifically, the major critical issue is given by the hydrophobicity of the EO, which is chemically in contrast with the hydrophilicity of the culture medium [26,27], leading the EO to work in unfavorable conditions. This problem is overcome in the broth microdilution test as the EO is solubilized in the broth culture with the aid of a surfactant. Furthermore, in the agar diffusion test, it is not possible to extrapolate the quantity of EO capable of inhibiting the growth of the microbial strain with absolute certainty as this quantity is strongly influenced by the ability of the EO to diffuse through the hydrophilic solid agar. Finally, the volatility of the EO compounds inevitably influence the correct determination of the MIC value [25,27].

Even if the broth microdilution test overcomes the bias of the solubilization of EOs, it is also crucial to consider the variability given by the type of surfactant used to solubilize the EO. In fact, the same analysis performed using three different vehicles against a resistant and a sensitive strain belonging to each microbial group shows how the surfactant influences the efficacy of CC-EO. Data reported in Table 4 unequivocally indicate that Tween80 improves the activity of CC-EO. Specifically, MIC_average_ values are statistically lower in five out of seven groups when using Tween80 instead of DMSO. Similarly, four out of seven groups show lower MIC_average_ values using Tween80 instead of ethanol. These findings suggest that Tween80 improves the solubilization and dispersion of CC-EO, potentially increasing its bioavailability and interaction with microbial cells. While the differences may not always be statistically significant, the overall trend supports its selection as the optimal vehicle.

These data agree with what was highlighted by Van de Vel E. et al. [28] regarding the ability of the vehicle to influence the antimicrobial activity of the EO. The data in Table 2 and Table 3 show that it is not possible to identify a conversion factor which allows vehicles to be interchanged. Therefore, an important question arises: “Which is the best EOs vehicle to use in the broth microdilution test?”. We believe it is crucial to evaluate the effectiveness of EOs under optimal conditions that maximize their antimicrobial action. This is because these tests are currently used in the microbiological routine to support the therapeutic choices of aromatherapy specialists. Therefore, it is important to determine the effective dose accurately to prevent the administration of potentially harmful dosages.

The antimicrobial activity of CC-EO vapors is highlighted in Table 5. The results of the microatmosphere test show the same efficacy as the other investigation methods. Specifically, the effectiveness is greater against *C. albicans* and GRAM+ strains (*S. aureus* and *S. pyogenes*), while it is weak against all GRAM−, with a total ineffectiveness against *E. faecalis*, *K. pneumoniae*, and *P. aeruginosa*.

Collectively, the in vitro sensitivity data suggest potential topical aromatherapy applications for CC-EO and its vapors in the management of pathologies, particularly those caused by GRAM+ bacteria or *C. albicans*, such as oral cavity disorders.

## 4. Materials and Methods

### 4.1. Strains and Growth Media

To develop experimental tests, a collection of 70 microbial strains belonging to positive clinical cultures were isolated. Specifically, 10 strains for each of the following species were studied: *S. aureus*, *E. coli*, *E. faecalis*, *K. pneumoniae*, *P. aeruginosa*, *S. pyogenes*, and *C. albicans*. To identify each strain, the MALDI-TOF mass-spectrometry method was used. The phenotypic antimicrobial susceptibility testing was performed using VITEK^®^ 2 system (Bio-Mérieux, Craponne, France). To perform the susceptibility categorization, European Committee on Antimicrobial Susceptibility Testing (EUCAST) breakpoint tables, version 15, were used [29]. Strains were stored in 20% glycerol stocks at −80 °C and cultured in Muller–Hinton broth (Sigma-Aldrich, Burlington, MA, USA) at 37 °C or Sensititre ™ YeastOne Broth (ThermoFisher Scientific, Waltham, MA, USA) at 30 °C. For all bacteria species, Muller–Hinton agar (Sigma-Aldrich, Burlington, MA, USA) was used. Whereas a Mueller–Hinton-F agar medium with 5% horse blood + β-NAD (β-nicotinamide adenine dinucleotide) for *Streptococcus* species and BCG agar (MEUS, Piove di Sacco, Italy) for *Candida albicans* was used.

### 4.2. Essential Oil

*C. carvi* essential oil (CC-EO), kindly provided by Pranarôm International, together with the related quality analysis (Avenue des Artisans, Ghislenghien, Belgium), was used.

### 4.3. Gas Chromatography–Mass Spectrometry

The chemical characterization of CC-EO was carried out using a Clarus 500 model gas chromatograph (Perkin Elmer, Waltham, MA, USA) equipped with a flame detector ionization (FID) and coupled with a mass spectrometer. A Varian Factor Four VF-5 capillary column was installed in the GC oven. The oven temperature was initially held at 60 °C and then increased at a rate of 6 °C/min to 250 °C, where it was maintained for 20 min. Helium was used as the carrier gas at a constant flow rate of 1 mL/min. The mass spectrometry (MS) conditions were as follows: the ion source temperature was set at 180 °C; the electron energy was 70 eV; the quadrupole temperature was maintained at 200 °C; the GC-MS interface was kept at 220 °C; and the scan range was from 40 to 550 mass units. Compound identification was achieved by comparing their mass spectra with those of pure components from the Wiley 2.2 and Nist 11 spectral libraries, as well as by comparing linear retention indices (LRIs), calculated using a series of alkane standards (C_8_–C_25_ *n*-alkanes), with literature values. The relative amounts of the identified components were expressed as percentages derived from FID peak-area normalization (mean of three replicates) without the use of an internal standard or any factor corrections.

### 4.4. Antimicrobial Activity of C. carvi Essential Oil

To evaluate the antimicrobial activity of CC-EO, three different microbial tests were developed. In particular, antibacterial and antifungal activity was evaluated using broth microdilution, disk diffusion, and microatmosphere methods [19].

#### 4.4.1. Broth Microdilution Assay

According to the EUCAST international guidelines, broth microdilution susceptibility tests were performed to evaluate minimum inhibitory concentration (MIC) values of *C. carvi* against the 70 strains. Briefly, using 96-well plates, 50 μL of a cell suspension (1 × 10^6^ CFU/mL for bacteria and 5 × 10^5^ UFC/mL for *C. albicans*) was added to an equal volume of broth in which a known concentration of EO was previously suspended. The antimicrobial activity of serial dilutions ranging from 72.8 µg/µL to 1.13 µg/µL was evaluated. To highlight the best vehicle of the CC-EO, Tween80, DMSO, and absolute ethanol, in a ratio 1:1 with the EO, were tested. The plates were incubated for 24 h at 37 °C. Two types of positive control are included: the growth control with the microorganism alone and the control with the vehicle only (i.e., without CC-EO). MIC values were visually determined and were defined as the lowest concentration with the total visual inhibition of growth compared with that of the positive controls. All tests were performed in biological and technical triplicate.

#### 4.4.2. Disk Diffusion Assay

According to the EUCAST international guidelines, to evaluate the antimicrobial activity of CC-EO, the disk diffusion method was also performed. A saline suspension with a density of 0.5 McFarland turbidity standard was prepared. Agar plates were inoculated by swabbing, in three directions, a sterile cotton swab dipped into the suspension. The inoculum was spread evenly over the entire agar surface, ensuring that there were no gaps between streaks. A 10 mm sterile filter paper disk impregnated with 20 µL (18.2 × 10^3^ µg) of pure EO or 20 µL of 7.2 × 10^3^ µg (corresponding to the highest quantity of EO tested in the microbroth dilution test) was placed in the center of the plate. The plates were sealed and incubated at 37 °C for 24 h under aerobic conditions. After this time, millimeters of the agar inhibition zone diameters (IZD) were measured. Positive controls (plates seeded with the sterile filter alone and with the vehicle only without the oil) were included. All tests were performed in biological and technical triplicate.

#### 4.4.3. Microatmosphere Assay

To evaluate the antimicrobial activity of CC-EO vapors, a microatmosphere assay was conducted. A 10 mm sterile filter paper disk, moistened with the maximum amount of CC-EO possible (50 μL equal to 4.5 × 10^4^ µg), was attached to the lid of a Petri dish containing the agar culture medium seeded, uniformly, with bacterial (1 × 10^7^ CFU/mL) or fungal (1 × 10^6^ CFU/mL) strains. The plates were reversed and placed on the upper lid containing the soaked filter paper and then sealed. The Petri dish, prepared as described, was incubated at 37 °C overnight. Positive controls without EO and with the vehicle only (i.e., without the oil) were used as references. All tests were performed in biological and technical triplicate.

### 4.5. Statistical Analysis

Normal distribution data were analyzed using mean and standard deviation parameters. Disk diffusion and microatmosphere test data were analyzed using two-way ANOVA followed by Šídák’s multiple comparisons test to assess statistical differences between treated and untreated samples. A *p*-value < 0.05 was considered statistically significant. All statistical analyses were performed using GraphPad Prism Verion 10.4.0 (621).

## 5. Conclusions

In recent years, antibiotic resistance has become a major threat to global health. Scientific research aimed at identifying novel therapeutic strategies to combat antimicrobial resistance is a critical priority. Natural substances represent an important reservoir of new therapeutic resources, and among these, EOs are known for their antimicrobial activity. To evaluate the effectiveness of an EO, it is important to choose the most appropriate microbiological test. Among the two tests most used in microbiological routines, the broth microdilution test has proven to be more suitable for EOs. Furthermore, the hydrophobic nature of these extracts requires the use of surfactants capable of dissolving them in the hydrophilic culture medium. Among the surfactants, the best seems to be Tween80, which is already widely used in the literature. CC-EO, obtained from *C. carvi* aromatic plant and its vapors, shows interesting antimicrobial activity against GRAM+ and *C. albicans*. Therefore, if the data are confirmed in in vivo studies, CC-EO could find application in the microbiological therapies of integrated medicine to reduce the development of antibiotic resistance.

## Figures and Tables

**Table 1:** Chemical composition (percentage mean value ± standard deviation) of *C. carvi* EO as determined by GC-MS.

N°	Component ^1^	LRI ^2^	LRI ^3^	%
1	sabinene	972	976	tr
2	β-myrcene	996	992	0.2 ± 0.02
3	limonene	1032	1028	31.9 ± 1.25
4	linalool	1105	1102	0.2 ± 0.02
5	trans-p-2,8-p-menthadien-1-ol	1124	1120	0.2 ± 0.02
6	limonene oxide	1142	1138	0.3 ± 0.02
7	cis-dihydrocarvone	1196	1194	0.9 ± 0.05
8	trans-dihydrocarvone	1199	1197	0.4 ± 0.03
9	carvone	1230	1226	65.1 ± 5.62
10	L-perillaldehyde	1238	1243	0.3 ± 0.02
11	methyl geranate	1310	1305	0.1 ± 0.01
12	cis-carvyl acetate	1350	1342	tr
13	α-copaene	1383	1388	tr
14	β-bourbonene	1395	1390	0.1 ± 0.01
15	β-caryophyllene	1431	1425	0.2 ± 0.02
16	caryophyllene oxide	1565	1570	0.1 ± 0.01
	SUM			100.0
	Monoterpenes			99.3
	Sesquiterpenes			0.4
	Others			0.3

^1^ The components are reported according to their elution order on apolar column. ^2^ Linear retention indices measured on apolar column. ^3^ Linear retention indices from the literature. tr: (mean value <0.1%).

**Table 2:** The table shows the minimum inhibitory concentration MIC values, MIC50, MIC90, and MIC average ± standard deviation (St. Dev.) (μg/μL) values of the tested microorganisms (*n* = 70) divided by species (10 strains each).

Minimum Inhibitory Concentration (MIC) (μg/μL)							
Strain ID	*S. aureus*	*E. coli*	*E. faecalis*	*K. pneumoniae*	*P. aeruginosa*	*S. pyogenes*	*C. albicans*
1	9.1 *	18.2	72.8	>72.8 *	72.8	1.1	4.6
2	18.2	18.2	72.8	72.8	>72.8	2.3	4.6
3	18.2	9.1 *	36.4	18.2 *	>72.8	2.3	4.6
4	72.8	36.4	18.2	18.2 *	>72.8 *	1.1	9.1
5	36.4	72.8 *	36.4	72.8 *	36.4	1.1	9.1
6	36.4	9.1	18.2	36.4 *	9.1	4.6	4.6
7	9.1 *	9.1 *	18.2	9.1	>72.8 *	9.1	4.6
8	9.1	18.2	9.1	18.2	72.8 *	9.1	4.6
9	72.8	4.55 *	72.8	2.3 *	>72.8	4.6	4.6
10	4.6	18.2	>72.8	72.8 *	>72.8	4.6	4.6
MIC50	18.2	18.2	36.4	18.2	>72.8	2.3	4.6
MIC90	72.8	36.4	72.8	72.8	>72.8	9.10	9.1
Average ± St. Dev.	28.7 ± 24.4	21.4 ± 19.1	N.A.	N.A.	N.A.	3.9 ± 2.9	5.5 ± 1.8

Note: The table reports only the mean MIC values due to the perfect reproducibility of the MIC values obtained in triplicates. N.A., not available. * MDR strains.

**Table 3:** IZD average ± standard deviation (St. Dev.), derived from biological and technical triplicates, of 70 microorganisms at two tested concentrations (7.3 × 10^3^ μg and 18.2 × 10^3^ μg) and IZD average ± standard deviation (St. Dev.).

Inhibition Zone Diameter (Average ± St. Dev.) (cm)							
Concentration (μg)	*S. aureus*	*E. coli*	*E. faecalis*	*K. pneumoniae*	*P. aeruginosa*	*S. pyogenes*	*C. albicans*
7.3 × 10^3^	1.2 ± 0.3	1.2 ± 0.2	0.0 ± 0.0	1.3 ± 0.1	0.0 ± 0.0	0.9 ± 0.8	3.0 ± 0.6
18.2 × 10^3^	4.5 ± 1.5	1.8 ± 0.4	1.6 ± 0.4	1.9 ± 0.5	0.0 ± 0.0	4.3 ± 1.0	8.0 ± 0.0

**Table 4:** The table shows the MIC values and MIC_average_ of R and S strains using Tween80, DMSO, and ethanol as EO vehicles in a broth microdilution assay.

Minimum Inhibitory Concentration (MIC) (μg/μL)								
	Strain ID	*S. aureus*	*E. coli*	*E. faecalis*	*K. pneumoniae*	*P. aeruginosa*	*S. pyogenes*	*C. albicans*
Tween80	R	9.1	4.6	72.8	72.8	72.8	2.3	4.6
	S	36.4	18.2	18.2	72.8	72.8	2.3	4.6
	Average ± St. Dev.	22.8 ± 19.3	11.4 ± 9.65	45.6 ± 38.6	72.8 ± 0.0	72.8 ± 0.0	2.28 ± 0.0	4.6 ± 0.0
DMSO	R	72.8	72.8	36.4	72.8	36.4	36.4	36.4
	S	72.8	>72.8	72.8	72.8	72.8	36.4	36.4
	Average ± St. Dev.	72.8 ± 0.0	N.A.	54.6 ± 23.0	72.8 ± 0.0	54.6 ± 20.9	36.4 ± 0.0	36.4 ± 0.0
Ethanol	R	72.8	72.8	36.4	72.8	36.4	18.2	18.2
	S	36.4	72.8	36.4	72.8	36.4	18.2	18.2
	Average ± St. Dev.	54.6 ± 20.9	72.8 ± 0.0	36.4 ± 0.0	72.8 ± 0.0	36.4 ± 0.0	18.2 ± 0.0	18.2 ± 0.0

Note: Not available (N.A.). R strains for *S. aureus*, *E. coli*, *K. pneumoniae*, and *P. aeruginosa* are MDR strains, and those for *E. faecalis*, *S. pyogenes*, and *C. albicans* are strains with the highest number of resistances in the AST among those available (Supplementary Materials Table S1).

**Table 5:** IZD (in centimeters) average ± standard deviation (St. Dev.), derived from biological and technical triplicates, of 70 microorganisms divided by species (10 strains each) at the tested concentration (4.5 × 10^4^ μg).

Inhibition Zone Diameter (Average ± SD) (cm)							
	*S. aureus*	*E. coli*	*E. faecalis*	*K. pneumoniae*	*P. aeruginosa*	*S. pyogenes*	*C. albicans*
Average ± St. Dev.	3.6 ± 0.7	2.2 ± 2.1	0.0 ± 0.0	0.0 ± 0.0	0.0 ± 0.0	3.0 ± 0.7	8.0 ± 0.0

## Data Availability

The original contributions presented in this study are included in the article/supplementary materials. Further inquiries can be directed to the corresponding author.

## Supplementary Materials

The following supporting information can be downloaded at https://www.mdpi.com/article/10.3390/ph18030321/s1, Table S1. Antimicrobial resistance of *S. aureus* (a), *E. coli* (b), *E. faecalis* (c), *P. aeruginosa* (d), *K. pneumoniae* (e), *S. pyogenes* (f) and *C. albicans* (g) clinical isolates; Table S2. Inhibition zone diameters (IZD, in centimeters) of 70 microorganisms at two tested concentrations (7.3 × 10^3^ µg and 18.2 × 10^3^ µg) and IZD Average ± Standard Deviation (St. Dev.), derived from biological and technical triplicates; Table S3. IZD (in centimeters) Average ± Standard Deviation (St. Dev.), derived from biological and technical triplicates, of 70 microorganisms divided by species (10 strains each) at tested concentration (4.5 × 10^4^ µg); Table S4: Analysis sheet on essential oil provided by the manufacturer.

## Abbreviations

The following abbreviations are used in this manuscript:

EOS	Essential oils
CC-EO	*Carum carvi* essential oil
LGT	Lateral gene transfer
MRSA	Methicillin-resistant *staphylococcus aureus*
MDR	Multidrug resistance
DMSO	Dimethyl sulfoxide
MALDI-TOF	Matrix-assisted laser desorption/ionization–time-of-flight mass spectrometry
AMR	Antimicrobial resistance
GC-MS	Gas chromatography–mass spectrometry
MIC	Minimum inhibitory concentration
IZD	Inhibition zone diameters
EMA	European medicines agency
AR-ISS	National surveillance of antibiotic resistance
