# Supplementary material for: In Vitro Study to Evaluate the Best Conditions Highlighting the Antimicrobial Activity of Carum carvi Essential Oil on Human Pathogen Isolates in Formulations Against the Spread of Antibiotic Resistance
Source: Pharmaceuticals (Basel). 2025 Feb 25;18(3):321. doi: 10.3390/ph18030321 (PMC11945957; doi:10.3390/ph18030321)
Supplement: Supplementary file 1 [file pharmaceuticals-18-00321-s001.zip › Supplementary material Table S4.pdf]

PRANARŌM international

# FICHE D'ANALYSE – ANALYSIS SHEET

## Huile essentielle – Essential oil

Nom botanique – botanical name: ***Carum carvi***
Nom commun – french name: CARVI
Numéro du lot – lot number: **OF46565**
Origine - origin: ---- PRANAROM - FINLANDE
Partie de la plante – part of the plant: FRUIT
Date de distillation – distillation date : 04/2019
Date de peremption – out of date : 05/2025

**Caractéristiques d'analyse – analysis characteristics:**

CPG – SM / CPG-FID AGILENT
Colonne : VF WAX 60-0,25-0,25
Programmation de température : 5 mn à 60 °C –2 °C/mn→250 °C – 5 mn à 250 °C
Gaz vecteur Hé : 23 psis

**Caractéristiques physiques** :

| | |
|---|---|
| Aspect – physical state | Liquide limpide |
| Couleur - color | Jaune très clair |
| Odeur - odour | Fraiche, épicée, caractéristique |
| Densité à 20°C - density | 0.915 |
| Densité à 15°C - density | 0.919 |
| Indice de réfraction à 20°C - refractive index | 1.487 4 |
| Pouvoir rotatoire à 20°C - optical rotation | + 77.90 ° |
| Miscibilité à l'éthanol à 80% - miscibility | 1.1 volumes d'alcool 80 % / 1 volume d'HE |
| Point d'éclair : SETAFLASH - flashpoint | 61.6 °C |

**Analyses pesticides – pesticide analysis :**

| | |
|---|---|
| **Pesticides Organochlorés : Dosage par GC MS détecteur XSD (méthode multirésidus interne validée selon la norme NF V03-110)**<br>**Liste des pesticides recherchés (Pharmacopée Européenne)**: Alachlor, Aldrine, Bromophos Ethyl, Bromophos Methyl, Chlordane, Chlorfenvinphos, Chlorpyriphos, Chlorpyriphos Methyl, Chlorthal Dimethyl, Cyfluthrine, Cyhalothrine lambda, Cypermethrine, Dichlofluanide, Dichlorvos, Dicofol (Kelthane), Dieldrine, Endosulfan, Endosulfan sulfate, Endrine, Fenchlorphos (Ronnel), Fenchlorphos-oxon, Fenvalerate, Fluvalinate, Heptachlor, Heptachlor epoxide, Hexachlorobenzene, Hexachlorocyclohexane α, Hexachlorocyclohexane β, Hexachlorocyclohexane δ, Hexachlorocyclohexane ε, Lindane, Methoxychlore, Mirex, Naled, o,p'-DDD , o,p'-DDE, o,p'-DDT, Oxychlordane, p,p'-DDD, p,p'-DDE, p,p'-DDT, Pentachloroaniline, Pentachloroanisole, Permethrine , Phosalone, Procymidone, Profenophos, Prothiofos, Quintozene, S421, Tecnazene, Tetradifon, Vinclozoline | **Résultats**<br>< LMR*<br>* Limite Maximale de Résidus autorisée |
| **Pesticides Organophosphorés : Dosage par GC MS détecteur FPD (méthode multirésidus interne validée selon la norme NF V03-110)**<br>**Liste des pesticides recherchés (Pharmacopée Européenne)**: Acephate, Azinphos Ethyl, Azinphos Méthyl, Bromophos Ethyl, Bromophos Méthyl, Chlorfenvinphos, Chlorpyriphos, Chlorpyriphos Methyl, Diazinon, Dichlorvos, Dimethoate, Ethion, Etrimphos, Fenchlorphos (Ronnel), Fenchlorphos-oxon, Fenitrothion, Fensulfothion (Dasanit), Fensulfothion-oxon, Fensulfothion-oxon-sulfone, Fensulfothion-sulfone, Fenthion, Fenthion-oxon, Fenthion-oxon-sulfone, Fenthion-oxon-sulfoxyde, Fenthion-sulfone, Fenthion-sulfoxyde, Fonofos, Malaoxon, Malathion, Mecarbam, Methacrifos, Methamidophos (Monitor), Methidathion, Monocrotophos, Naled, Omethoate, Paraoxon, Paraoxon Méthyl, Parathion Ethyl, Parathion Methyl, Phosalone, Phosmet, Pirimiphos Ethyl, Pirimiphos Méthyl, Profenophos, Prothiofos, Quinalphos. | **Résultats**<br>< LMR*<br>* Limite Maximale de Résidus autorisée |

# Profil CHROMATOGRAPHIQUE

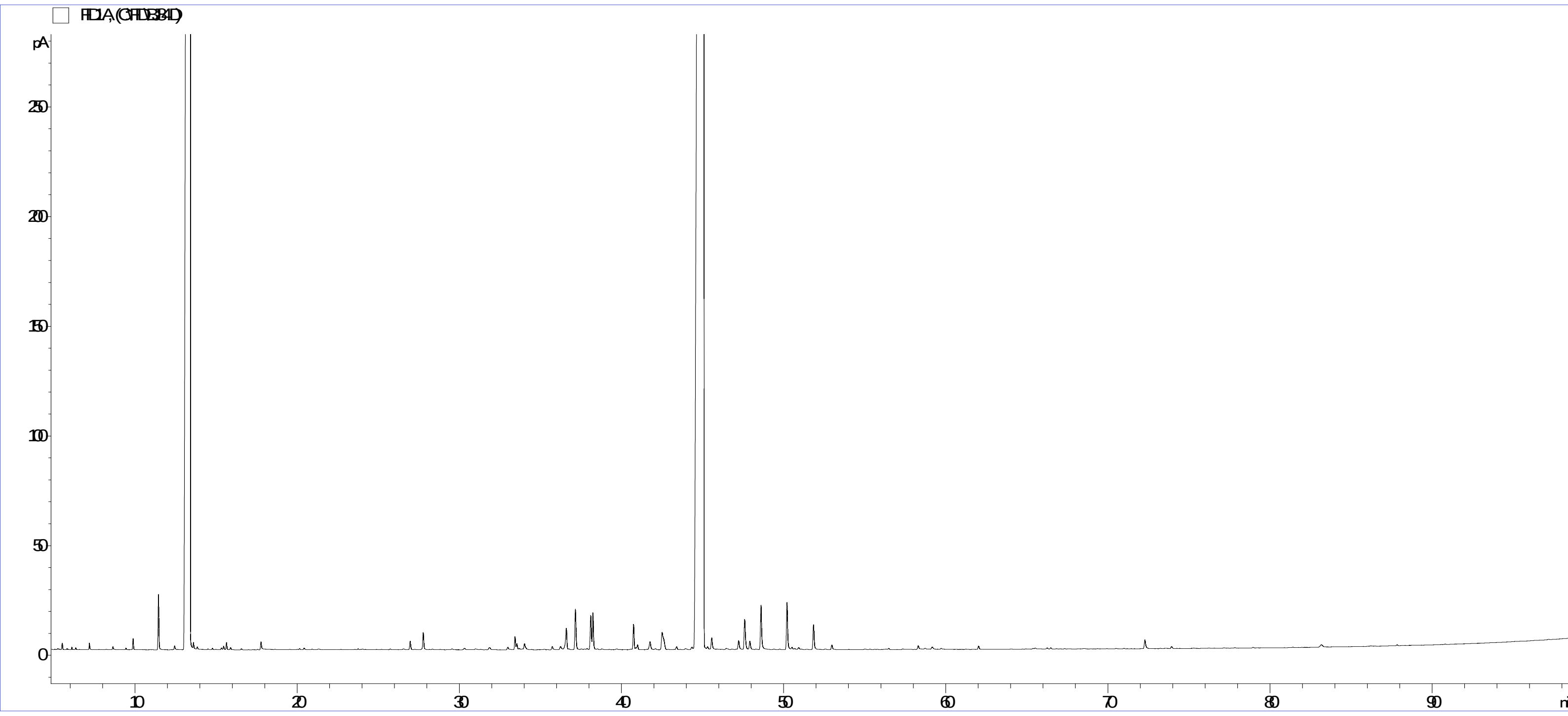

# Tableau de résultats 1 : CARAWAY

LOT OF46565

| Pics | Temps de rétention | Constituants | % |
|---|---|---|---|
| 1 | 4,7 | ACETONE | 0,02 |
| 2 | 5,4 | 2-METHYL BUTANAL | 0,01 |
| 3 | 5,5 | ISOVALERALDEHYDE | 0,02 |
| 4 | 6,0 | COMPOSÉ Mw=124 | 0,01 |
| 5 | 7,1 | alpha-PINENE | 0,02 |
| 6 | 7,2 | alpha-THUYENE | 0,01 |
| 7 | 8,6 | HEXANAL | 0,01 |
| 8 | 9,4 | beta-PINENE | 0,01 |
| 9 | 9,8 | SABINENE | 0,04 |
| 10 | 11,4 | beta-MYRCENE | 0,22 |
| 11 | 11,5 | alpha-PHELLANDRENE | 0,01 |
| 12 | 12,4 | HEPTANAL | 0,02 |
| 13 | 13,4 | **LIMONENE** | **39,95** |
| 14 | 13,6 | beta-PHELLANDRENE | 0,03 |
| 15 | 13,8 | MENTHATRIENE ISOMERE | 0,01 |
| 16 | 14,5 | 2-PENTYL FURANE | 0,01 |
| 17 | 14,7 | Cis-beta-OCIMENE | 0,01 |
| 18 | 15,3 | gamma-TERPINENE | 0,01 |
| 19 | 15,4 | MENTHATRIENE ISOMERE | 0,02 |
| 20 | 15,6 | Trans-beta-OCIMENE | 0,03 |
| 21 | 15,8 | 3-OCTANONE | 0,01 |
| 22 | 16,5 | p-CYMENE | 0,01 |
| 23 | 17,7 | OCTANAL | 0,04 |
| 24 | 20,1 | COMPOSÉ Mw=152 | 0,01 |
| 25 | 20,4 | 6-METHYL-5-HEPTEN-2-ONE | 0,01 |
| 26 | 26,5 | alpha-p-DIMETHYLSTYRENE | 0,01 |
| 27 | 26,9 | Cis-1,2-EPOXYDE DE LIMONENE | 0,04 |
| 28 | 27,7 | Trans-1,2-EPOXYDE DE LIMONENE | 0,09 |
| 29 | 29,5 | SESQUITERPENE | 0,01 |
| 30 | 30,3 | alpha-COPAENE | 0,01 |
| 31 | 31,8 | beta-BOURBONENE | 0,02 |
| 32 | 33,0 | 2-NONENAL | 0,02 |
| 33 | 33,4 | LINALOL | 0,06 |
| 34 | 33,5 | 8,9-cis-EPOXYDE DE LIMONENE | 0,03 |
| 35 | 34,0 | 8,9-trans-EPOXYDE DE LIMONENE | 0,04 |
| 36 | 35,7 | COMPOSÉ Mw=150 | 0,02 |
| 37 | 36,2 | COMPOSÉ Mw=150 | 0,02 |
| 38 | 36,6 | beta-CARYOPHYLLENE | 0,14 |
| 39 | 37,1 | Cis-DIHYDROCARVONE | 0,22 |
| 40 | 38,1 | Trans-DIHYDROCARVONE | 0,18 |
| 41 | 38,2 | Cis-p-MENTHA-2,8-DIEN-1-OL | 0,19 |
| 42 | 40,7 | Trans-p-MENTHA-2,8-DIEN-1-OL | 0,12 |
| 43 | 40,9 | alpha-HUMULENE | 0,01 |
| 44 | 41,0 | E-beta-FARNESENE | 0,03 |
| 45 | 41,7 | NERAL | 0,06 |
| 46 | 42,5 | alpha-TERPINEOL | 0,10 |
| 47 | 42,6 | GERANATE DE METHYLE | 0,09 |

# Tableau de résultats 2 : CARAWAY

LOT OF46565

| Pics | Temps de rétention | Constituants | % |
|---|---|---|---|
| 48 | 43,4 | GERMACRENE D | 0,02 |
| 49 | 44,4 | BICYCLOGERMACRENE | 0,02 |
| 50 | 44,9 | **CARVONE** | **56,57** |
| 51 | 45,5 | Trans-PIPERITOL | 0,08 |
| 52 | 47,2 | Cis-DIHYDROCARVEOL | 0,06 |
| 53 | 47,6 | PERILLALDEHYDE | 0,19 |
| 54 | 47,9 | Trans-DIHYDROCARVEOL | 0,05 |
| 55 | 48,6 | Trans-CARVEOL | 0,26 |
| 56 | 50,2 | Trans-ANETHOL + OXYDE DE CARVONE | 0,27 |
| 57 | 50,5 | ACIDE CAPROÏQUE | 0,02 |
| 58 | 50,9 | GERANIOL | 0,01 |
| 59 | 51,8 | Cis-CARVEOL | 0,15 |
| 60 | 52,9 | Cis-p-MENTHA-1,8-DIENE-2-OL | 0,03 |
| 61 | 56,5 | ACIDE HEPTANOÏQUE | 0,01 |
| 62 | 58,3 | OXYDE DE CARYOPHYLLENE | 0,02 |
| 63 | 59,1 | ALCOOL PERILLIQUE | 0,02 |
| 64 | 62,0 | ACIDE CAPRYLIQUE | 0,02 |
| 65 | 65,5 | SPATHULENOL | 0,01 |
| 66 | 66,2 | TRIMETHYL PENTADECANONE | 0,01 |
| 67 | 66,5 | COMPOSÉ CETONIQUE | 0,01 |
| 68 | 72,2 | Trans-LIMONENE-1,2-DIOL | 0,06 |
| 69 | 73,9 | MATRICARIA ESTER | 0,01 |
| 70 | 83,1 | COMPOSÉ AROMATIQUE | 0,03 |
| | | **TOTAL** | **99,99** |

Date de l'analyse – date of the analysis : Mai 2020,

Pranarôm Int.
C. Schulze
Contrôle qualité
