# Supplementary material for: In Vitro Study to Evaluate the Best Conditions Highlighting the Antimicrobial Activity of Carum carvi Essential Oil on Human Pathogen Isolates in Formulations Against the Spread of Antibiotic Resistance
Source: Pharmaceuticals (Basel). 2025 Feb 25;18(3):321. doi: 10.3390/ph18030321 (PMC11945957; doi:10.3390/ph18030321)
Supplement: Supplementary file 1 [file pharmaceuticals-18-00321-s001.zip › Supplementary materials Tables S1-S3.pdf]

Supplementary Materials

Table S1. Antimicrobial resistance of *S. aureus* (a), *E. coli* (b), *E. faecalis* (c), *P. aeruginosa* (d), *K. pneumoniae* (e), *S. pyogenes* (f) and *C. albicans* (g) clinical isolates.

a

S. aureus	**PEN**	**OX**	**CPT**	**GEN**	**LVX**	**ERY**	**CLI**	**LZD**	**DAP**	**TEC**	**VAN**	**TET**	**TGC**	**FUS**	**MUP**	**RIF**	**SXT**
SA.1 (MDR)	R	R	S	R	I	S	S	S	S	S	S	S	S	S	S	S	S
SA.2	S	S	S	S	I	R	R	S	S	S	S	S	S	S	S	S	S
SA.3	R	S	S	S	I	S	S	S	S	S	S	S	S	S	S	S	S
SA.4	R	S	S	S	I	S	S	S	S	S	S	S	S	S	S	S	S
SA.5	R	S	S	S	I	S	S	S	S	S	S	S	S	S	S	S	S
SA.6	S	S	S	S	I	S	S	S	S	S	S	S	S	S	S	S	S
SA.7 (MDR)	R	R	S	R	I	S	S	S	S	S	S	S	S	S	S	S	S
SA.8	R	S	S	S	I	S	S	S	S	S	S	S	S	S	S	S	S
SA.9	S	S	S	S	I	S	S	S	R	R	S	S	S	S	S	S	S
SA.10	R	S	S	S	I	S	S	S	S	S	S	S	S	S	S	S	S

b

E. coli	**AMC**	**TZP**	**CTX**	**CAZ**	**CZA**	**C/T**	**FEP**	**IPM**	**MEM**	**AMK**	**GEN**	**TOB**	**CIP**	**CST**	**SXT**
EC.1	R	S	S	S	S	S	S	S	S	S	S	S	S	S	S
EC.2	S	S	R	R	S	S	I	S	S	S	S	S	R	S	S
EC.3 (MDR)	R	S	R	R	S	S	R	S	S	S	R	R	R	S	R
EC.4	S	S	S	S	S	S	S	S	S	S	S	S	S	S	S
EC.5 (MDR)	R	S	R	R	S	S	R	S	S	S	R	R	R	S	R
EC.6	S	S	S	S	S	S	S	S	S	S	S	S	S	S	S
EC.7 (MDR)	R	R	R	R	S	R	R	S	S	S	R	R	I	S	R
EC.8	S	S	R	I	S	S	I	S	S	S	S	S	R	S	S
EC.9 (MDR)	R	S	R	R	S	S	R	S	S	S	R	R	R	S	R
EC.10	R	S	S	S	S	S	S	S	S	S	S	S	I	S	S

c

E. faecalis	**AMP**	**AMC**	**SAM**	**IPM**	**HLR-KEN**	**HLR-STR**	**CIP**	**LEV**	**LNZ**	**TEC**	**VAN**	**TGC**	**NIT**
EF.1	S	S	S	I	S	S	S	S	S	S	S	S	S
EF.2	S	S	S	I	R	S	R	R	S	S	S	S	S
EF.3	S	S	S	I	R	S	S	S	S	S	S	S	S
EF.4	S	S	S	I	S	S	S	S	S	S	S	S	S
EF.5	S	R	S	I	R	S	S	S	S	S	S	S	S
EF.6	S	S	S	I	S	S	S	S	S	S	S	S	S
EF.7	S	S	S	I	R	S	S	S	S	S	S	S	S
EF.8	S	S	S	I	S	S	S	S	S	S	S	S	S
EF.9	S	S	S	I	R	R	S	S	S	S	S	S	S
EF.10	S	S	S	I	S	S	S	S	S	S	S	S	S

d

P. aeruginosa	TZP	CAZ	CZA	C/T	FEP	IPM	MEM	AMK	TOB	CIP	CST
PA.1	I	I	S	S	I	I	S	S	S	R	S
PA.2	I	I	S	S	I	I	S	S	S	I	S
PA.3	I	I	S	S	I	R	I	S	S	I	S
PA.4$^{(MDR)}$	R	I	S	S	I	R	I	S	S	R	S
PA.5	I	I	S	S	I	I	S	S	S	R	S
PA.6	R	I	S	S	I	I	S	S	S	I	S
PA.7$^{(MDR)}$	R	I	S	S	R	R	R	S	S	R	S
PA.8$^{(MDR)}$	R	R	R	R	R	R	R	R	R	R	S
PA.9	R	R	S	S	R	I	S	S	S	I	S
PA.10	I	I	S	S	I	R	I	S	S	I	S

e

K. pneumoniae	AMC	TZP	CTZ	CAZ	CZA	C/T	FEP	IPM	MEM	AMK	GEN	TOB	CIP	CST	SXT
KP.1$^{(MDR)}$	R	R	R	R	S	S	R	S	S	S	R	R	R	S	R
KP.2	S	S	S	S	S	S	S	S	S	S	S	S	S	S	S
KP.3$^{(MDR)}$	R	R	S	I	S	S	I	S	S	S	S	S	R	S	S
KP.4$^{(MDR)}$	R	R	R	R	S	S	R	S	S	S	R	R	R	S	R
KP.5$^{(MDR)}$	R	R	R	I	S	S	R	S	S	S	S	S	S	S	S
KP.6$^{(MDR)}$	R	R	R	R	S	S	R	S	S	S	S	R	R	S	R
KP.7	R	S	S	S	S	S	S	S	S	S	S	S	S	S	R
KP.8	S	S	S	S	S	S	S	S	S	S	S	S	S	S	S
KP.9$^{(MDR)}$	R	R	R	R	S	R	R	R	R	S	S	S	R	S	R
KP.10$^{(MDR)}$	R	R	R	R	R	R	R	R	R	S	R	R	R	S	R

f

S. pyogenes	CLI	CHL	ERY	LEV	LNZ	MXF	PEN	TEC	TET	SXT	VAN
SP.1	S	S	S	I	S	S	S	S	S	S	S
SP.2	S	S	R	I	S	S	S	S	S	S	S
SP.3	S	S	S	I	S	S	S	S	S	S	S
SP.4	S	S	S	I	S	S	S	S	S	S	S
SP.5	S	S	S	I	S	S	S	S	S	S	S
SP.6	S	S	S	I	S	S	S	S	S	S	S
SP.7	S	S	S	I	S	S	S	S	S	S	S
SP.8	S	S	S	I	S	S	S	S	S	I	S
SP.9	S	S	S	I	S	S	S	S	S	S	S
SP.10	S		S	I	S	S	S	S	S	I	S

Note. *S. aureus* (SA), *E. coli* (EC), *E. faecalis* (EF), *P. aeruginosa* (PA), *K. pneumoniae* (KP), *S. pyogenes* (SP), Amoxicillin-Clavulanate (AMC), Amikacin (AMK), Ampicillin (AMP), Ceftazidime (CAZ), Chloramphenicol (CHL), Ciprofloxacin (CIP), Clindamycin (CLI), Cefepime-Tazobactam (CPT), Cefotaxime (CTZ), Ceftolozane-Tazobactam (C/T), Ceftazidime-Avibactam (CZA), Colistin (CST), Daptomycin (DAP), Erythromycin (ERY), Cefepime (FEP), Cefoxitin (FOX), Fusidic Acid (FUS), Gentamicin (GEN), High-Level Resistance Gentamicin (HLR-GEN), High-Level Resistance Kanamycin (HLR-KEN), High-Level Resistance Streptomycin

(HLR-STR), Imipenem (IPM), Kanamycin (KEN), Levofloxacin (LEV), Linezolid (LNZ), Levofloxacin (LVX), Linezolid (LZD), Meropenem (MEM), Mupirocin (MUP), Moxifloxacin (MXF), Nitrofurantoin (NIT), Oxacillin (OX), Penicillin (PEN), Rifampin (RIF), Ampicillin-Sulbactam (SAM), Trimethoprim-Sulfamethoxazole (SXT), Teicoplanin (TEC), Tetracycline (TET), Tigecycline (TGC), Tobramycin (TOB), Piperacillin-Tazobactam (TZP), Vancomycin (VAN), Resistant (R), Susceptible (S) and Intermediate (I).

g

C. albicans	AMP-B	FLU	ITR	VOR	POS	MFG	CAS	ANI
CA.1	S	S	S	S	S	S	S	S
CA.2	S	S	S	S	S	S	S	S
CA.3	S	S	S	S	S	S	S	S
CA.4	S	S	S	S	S	S	S	S
CA.5	S	R	R	R	R	S	S	R
CA.6	S	S	S	S	S	S	S	S
CA.7	S	S	S	S	S	S	S	S
CA.8	S	S	S	S	S	S	S	S
CA.9	S	R	R	R	R	S	S	R
CA.10	S	S	S	S	S	S	S	S

Note. *C. albicans* (CA), Anidulafungin (ANI), Amphotericin B (AMP-B), Caspofungin (CAS), Fluconazole (FLU), Itraconazole (ITR), Isavuconazole (ISA), Micafungin (MFG), Posaconazole (POS), Voriconazole (VOR), Resistant (R) and Susceptible (S).

Table S2. Inhibition zone diameters (IZD, in centimeters) of 70 microorganisms at two tested concentrations (7.3 × 10^3 μg and 18.2 × 10^3 μg) and IZD Average ± Standard Deviation (St. Dev.), derived from biological and technical triplicates.

		Inhibition Zone Diameter ± SD (cm)						
**Strain ID**	**Concentration (μg)**	***S. aureus***	***E. coli***	***E. faecalis***	***K. pneumoniae***	***P. aeruginosa***	***S. pyogenes***	***C. albicans***
1	7.3 × 10^3	1.1 ± 0.1	1.2 ± 0.1	0.0 ± 0.0	1.4 ± 0.2	0.0 ± 0.0	1.7 ± 0.3	3.4 ± 0.3
	18.2 × 10^3	5.9 ± 1.4	1.9 ± 0.2	1.7 ± 0.3	2.1 ± 0.6	0.0 ± 0.0	4.6 ± 0.5	8.0 ± 0.0
2	7.3 × 10^3	1.1 ± 0.0	1.1 ± 0.0	0.0 ± 0.0	1.4 ± 0.0	0.0 ± 0.0	1.8 ± 0.3	3.2 ± 0.2
	18.2 × 10^3	4.4 ± 1.8	1.5 ± 0.6	0.0 ± 0.0	1.9 ± 0.5	0.0 ± 0.0	3.9 ± 0.3	8.0 ± 0.0
3	7.3 × 10^3	1.1 ± 0.0	1.2 ± 0.1	0.0 ± 0.0	1.3 ± 0.1	0.0 ± 0.0	2.2 ± 0.2	2.5 ± 0.3
	18.2 × 10^3	5.5 ± 1.8	1.8 ± 0.2	1.8 ± 0.2	2.1 ± 0.3	0.0 ± 0.0	4.9 ± 0.8	8.0 ± 0.0
4	7.3 × 10^3	1.1 ± 0.1	1.1 ± 0.0	0.0 ± 0.0	1.3 ± 0.1	0.0 ± 0.0	0.0 ± 0.0	2.6 ± 0.2
	18.2 × 10^3	3.3 ± 0.6	1.8 ± 0.2	1.8 ± 0.2	1.8 ± 0.3	0.0 ± 0.0	5.2 ± 0.2	8.0 ± 0.0
5	7.3 × 10^3	1.3 ± 0.1	1.2 ± 0.1	0.0 ± 0.0	1.3 ± 0.1	0.0 ± 0.0	1.6 ± 0.7	3.0 ± 0.6
	18.2 × 10^3	3.6 ± 1.2	1.8 ± 0.2	1.9 ± 0.5	1.9 ± 0.1	0.0 ± 0.0	4.8 ± 0.2	8.0 ± 0.0
6	7.3 × 10^3	1.1 ± 0.0	1.2 ± 0.1	0.0 ± 0.0	1.3 ± 0.2	0.0 ± 0.0	0.0 ± 0.0	2.3 ± 0.2
	18.2 × 10^3	2.8 ± 0.4	1.6 ± 0.1	1.9 ± 0.1	1.8 ± 0.5	0.0 ± 0.0	6.1 ± 0.3	8.0 ± 0.0
7	7.3 × 10^3	1.1 ± 0.0	1.1 ± 0.2	0.0 ± 0.0	1.3 ± 0.1	0.0 ± 0.0	1.2 ± 0.1	3.5 ± 0.4
	18.2 × 10^3	4.9 ± 1.3	1.8 ± 0.2	1.6 ± 0.3	1.7 ± 0.4	0.0 ± 0.0	3.5 ± 0.1	8.0 ± 0.0
8	7.3 × 10^3	1.4 ± 0.2	1.2 ± 0.1	0.0 ± 0.0	1.2 ± 0.1	0.0 ± 0.0	0.0 ± 0.0	3.2 ± 0.3
	18.2 × 10^3	4.4 ± 0.0	1.6 ± 0.1	1.7 ± 0.2	2.1 ± 0.8	0.0 ± 0.0	3.0 ± 0.1	8.0 ± 0.0
9	7.3 × 10^3	1.9 ± 0.2	1.5 ± 0.4	0.0 ± 0.0	1.3 ± 0.1	0.0 ± 0.0	0.0 ± 0.0	4.0 ± 0.2
	18.2 × 10^3	6.4 ± 0.5	2.3 ± 0.5	1.5 ± 0.4	1.2 ± 0.1	0.0 ± 0.0	4.2 ± 0.3	8.0 ± 0.0
10	7.3 × 10^3	1.1 ± 0.1	1.1 ± 0.1	1.1 ± 0.1	1.4 ± 0.3	0.0 ± 0.0	0.0 ± 0.0	2.9 ± 0.2
	18.2 × 10^3	3.7 ± 0.6	2.3 ± 0.1	1.5 ± 0.4	2.0 ± 0.4	0.0 ± 0.0	3.1 ± 0.4	8.0 ± 0.0
Average ± St. Dev.	7.3 × 10^3	1.2 ± 0.3	1.2 ± 0.2	0.0 ± 0.0	1.3 ± 0.1	0.0 ± 0.0	0.9 ± 0.8	3.0 ± 0.6
	18.2 × 10^3	4.5 ± 1.5	1.8 ± 0.4	1.6 ± 0.4	1.9 ± 0.5	0.0 ± 0.0	4.3 ± 1.0	8.0 ± 0.0

Table S3. IZD (in centimeters) Average ± Standard Deviation (St. Dev.), derived from biological and technical triplicates, of 70 microorganisms divided by species (10 strains each) at tested concentration (4.5×10^4 μg).

	Inhibition Zone Diameter ± SD (cm)						
**Strain ID**	***S. aureus***	***E. coli***	***E. faecalis***	***K. pneumoniae***	***P. aeruginosa***	***S. pyogenes***	***C. albicans***
1	3.4 ± 0.3	0.0 ± 0.0	0.0 ± 0.0	0.0 ± 0.0	0.0 ± 0.0	3.2 ± 0.2	8.0 ± 0.0
2	3.6 ± 0.4	0.0 ± 0.1	0.0 ± 0.0	0.0 ± 0.0	0.0 ± 0.0	3.8 ± 0.2	8.0 ± 0.0
3	3.9 ± 0.2	8.0 ± 0.0	0.0 ± 0.0	0.0 ± 0.0	0.0 ± 0.0	3.5 ± 0.3	8.0 ± 0.0
4	2.9 ± 0.5	1.5 ± 0.2	0.0 ± 0.0	0.0 ± 0.0	0.0 ± 0.0	3.4 ± 0.4	8.0 ± 0.0
5	3.9 ± 0.6	2.3 ± 0.4	0.0 ± 0.0	0.0 ± 0.0	0.0 ± 0.0	4.1 ± 0.3	8.0 ± 0.0
6	2.3 ± 0.3	1.5 ± 0.3	0.0 ± 0.0	0.0 ± 0.0	0.0 ± 0.0	2.8 ± 0.3	8.0 ± 0.0
7	3.6 ± 0.3	1.2 ± 0.2	0.0 ± 0.0	0.0 ± 0.0	0.0 ± 0.0	2.6 ± 0.6	8.0 ± 0.0
8	3.4 ± 0.7	2.0 ± 0.5	0.0 ± 0.0	0.0 ± 0.0	0.0 ± 0.0	2.3 ± 0.3	8.0 ± 0.0
9	4.3 ± 0.6	3.2 ± 0.7	0.0 ± 0.0	0.0 ± 0.0	0.0 ± 0.0	3.0 ± 0.4	8.0 ± 0.0
10	4.5 ± 0.4	1.6 ± 0.1	0.0 ± 0.0	0.0 ± 0.0	0.0 ± 0.0	1.8 ± 0.6	8.0 ± 0.0
Average ± St. Dev.	3.6 ± 0.7	2.2 ± 2.1	0.0 ± 0.0	0.0 ± 0.0	0.0 ± 0.0	3.0 ± 0.7	8.0 ± 0.0
